# An investigation of the mechanical and microstructural evolution of a TiNbZr alloy with varied ageing time

**DOI:** 10.1038/s41598-018-24155-y

**Published:** 2018-04-10

**Authors:** Arne Biesiekierski, Jixing Lin, Khurram Munir, Sertan Ozan, Yuncang Li, Cuie Wen

**Affiliations:** 1 School of Engineering, RMIT University, Melbourne, Victoria 3001 Australia; 2 Advanced Material Research and Development Center, Zhejiang Industry & Trade Vocational College, Wenzhou, Zhejiang 325003 China; 3 Department of Mechanical Engineering, Bozok University, Yozgat, 66100 Turkey

## Abstract

Alloys comprised of the highly biocompatible elements titanium, niobium and zirconium have been a major focus in recent years in the field of metallic biomaterials. To contribute to the corpus of data in this field, the current paper presents results from a thorough microstructural and mechanical investigation of Ti-32Nb-6Zr subjected to a variety of ageing treatments. The presented alloy was stabilized to the higher temperature, body-centred cubic phase, showing only minimal precipitation on prolonged ageing, despite the presence of nanoscaled spinodal segregation arising from the Nb-Zr interaction. It further showed excellent mechanical properties, with tensile yield stresses as high as 820 MPa and Young’s moduli as low as 53 GPa. This leads to the ratio of strength to modulus, also known as the admissible strain, reaching a maximum of 1.3% after 6 hours ageing. These results are further supported by similar measurements from nanoindentation analysis.

## Introduction

The field of biomedical alloys is constantly evolving, with many novel alloy compositions arising in the last two decades in particular. Further work is ongoing still, with particular focus dedicated to the alloys of titanium (Ti), due to this element’s highly desirable mechanical and biological properties^1–3^.

Along with the alloying elements of Nb and Zr, likewise supremely biocompatible metals^3^, this composition-space allows for the development of strong, lightweight, and long-life alloys that may also display a low modulus; this latter property is desirable given the existing concerns over stress-shielding in orthopaedic implants, which arise due to mismatch in the Young’s modulus (*E*) of the implant with the stiffness of the surrounding bone^4^. While the stiffness of an implant can be reduced by rendering it porous, that comes with the cost of weakening the resulting material^5^. For this reason, a value known as the admissible strain, the ratio of the strength to the elastic modulus, is of particular interest in the design of these materials, with a higher admissible strain allowing for a greater range of achievable stiffness and strength profiles in the final implant.

**Figure 1:**
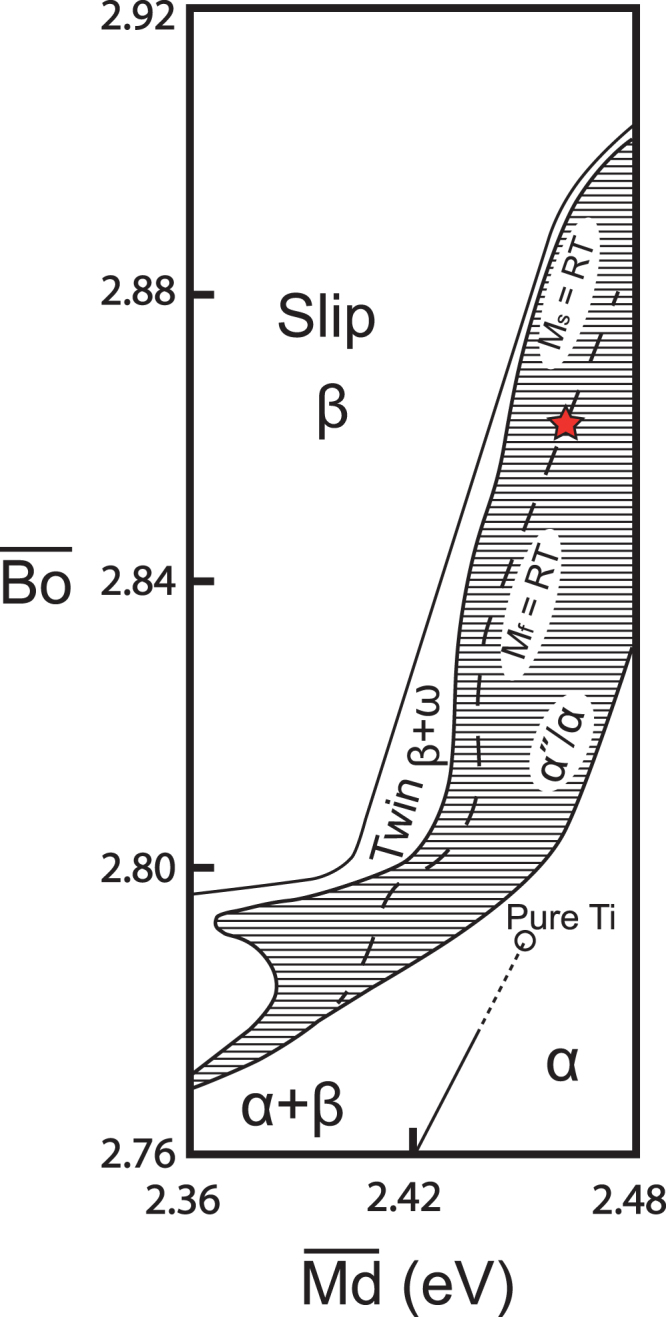
Electronic parameter phase map reconstructed from information in^7–10^. Nominal composition is indicated via red star.

The most promising mechanical and functional abilities in this regard are expected to be obtained in those areas where the body-centred-cubic (BCC), titanium β-phase is minimally stable^6^. However, given the large composition-space that these novel alloys can represent, determining the region that fits this for a given alloy may be difficult. One solution to this end is through the use of phase diagrams, as in Fig. 1, built with consideration of three general electronic parameters, which have shown great promise in the design and development of Ti-based alloys^7–10^; the mean electron/atom ($\overline{e/a}$) ratio, the mean bond order ($\overline{Bo}$) and the mean d-orbital energy level ($\overline{Md}$) given in eV. However, while the use of phase diagrams allow for a more general solution, these phase diagrams present information for alloys in the solution-treated and quenched state, and say little about the ageing response of the alloys.

In addition to this, these phase diagrams are further complicated by the possible presence of spinodal decomposition, which may result from the interaction of certain alloying compounds; of particular note are the elements Ta, Nb, and Zr, which are widely used in alloys of these types due to their beneficial impact on both the microstructural and biological properties of β-phase Ti alloys^3^.

As such, this study attempts to provide a systematic investigation of the microstructural and mechanical properties of an alloy in the region of interest in response to ageing treatment. To this end, a composition displaying a meta-stable β-phase, and composed of elements susceptible to spinodal decomposition was investigated under a range of ageing conditions. In addition to providing information about ageing response of alloys in this composition space, it is hoped that this work will also provide a useful resource for comparison of mechanical and microstructural properties with similar alloys.

## Results

### Microstructural Analysis

**Figure 2:**
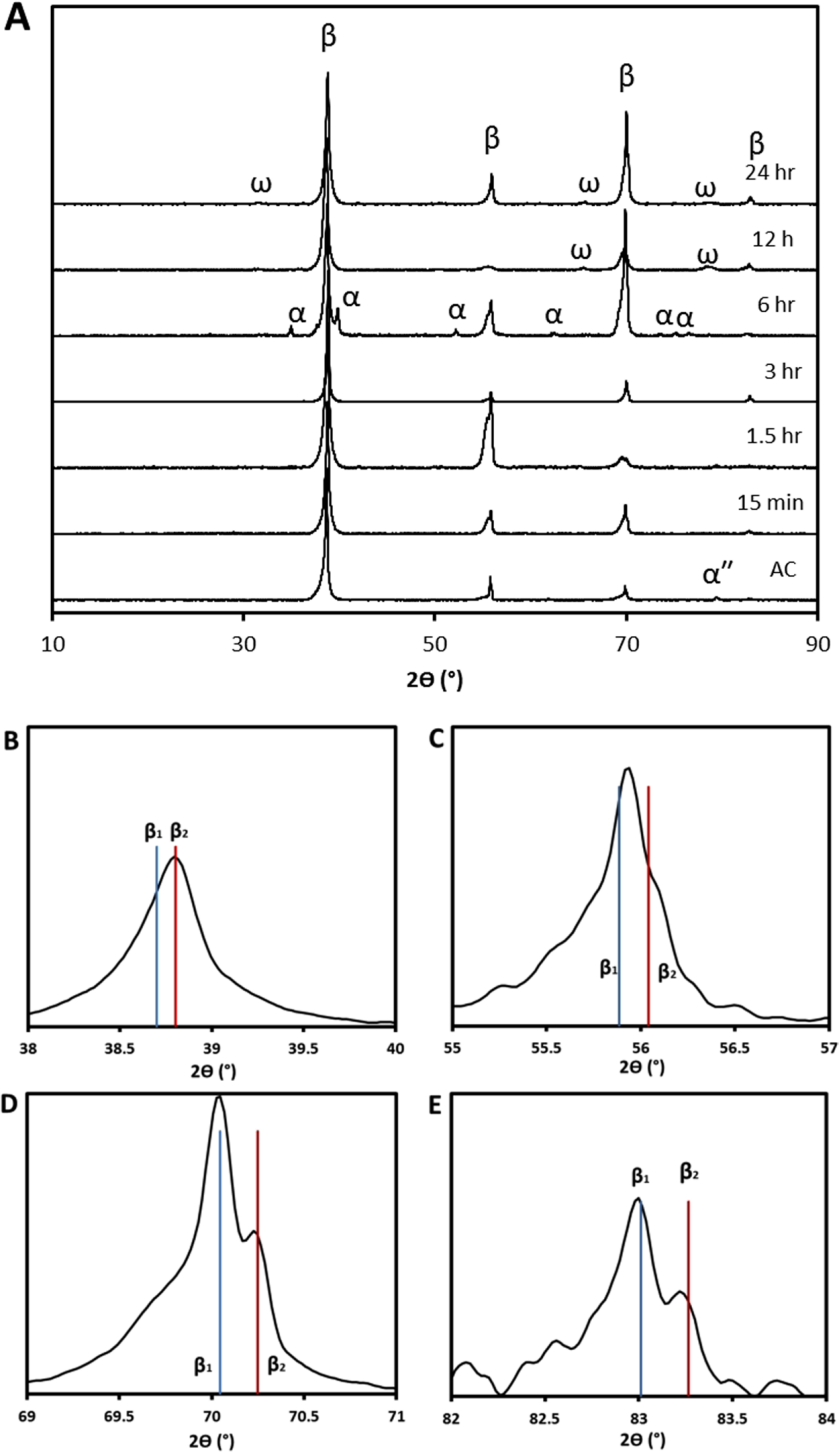
XRD spectra and measured lattice parameters. (**A**) XRD spectra (**B**–**E**) Expanded view of {110}β (**B**), {200}β (**C**), {211}β (**D**) and {220}β (**E**) peaks from 24 hr condition. Vertical lines in B-E included to show estimated *Im-3m* peak maxima. Vertical axes of all subfigures represent relative intensity.

Spectra from X-ray diffraction (XRD) analysis are given in Fig. 2A. It is immediately apparent that an *Im-3m* BCC lattice, analogous to the Ti β-phase and consistent with that expected for the Ti-Nb-Zr system, dominates all ageing conditions^11^. This phase showed an average lattice parameter of 3.30 ± 0.01 Å across the various ageing conditions, consistent with that expected for the BCC Ti and Nb phases. In addition to this, certain other phases can be resolved depending on the ageing condition; the most pronounced are peaks consistent with a *P6/mmm* lattice analogous to the Ti-ω phase, which was observed in the 12- and 24 hr ageing conditions. The indexed peaks closely obeyed the known lattice parameter relations between these phases, ${a}_{\omega }=\sqrt{2}{a}_{\beta }$ and ${c}_{\omega }=\frac{\sqrt{3}}{2}{a}_{\beta }$^12^, suggesting minimal composition difference between these phases.

In addition to this, the 6 hour state showed additional from a *P63/mmc* hexagonal phase equivalent to the Ti-α phase, with lattice parameters a_α_ = 2.97 and c_α_ = 4.76. Finally, a further single peak was observed at 79.6° in the AC material. While it is impossible to conclusively identify a phase from a single peak, this peak coincides with that expected for the {041} reflection of the orthorhombic *Cmcm* α″ phase, and so is tentatively indexed as such.

Although no additional peaks corresponding to distinct space-groups were noted, one further feature of interest could be resolved throughout all ageing conditions; peaks corresponding to the β-phase displayed some degree of peak splitting upon close inspection, examples of which are given from the 24 hr condition in Fig. 2B–E. While this splitting was not resolvable in the {110}_*β*_ maxima due to insufficient angular resolution (such as in Fig. 2B), this splitting/asymmetry was observed in all other peaks under at least one aging condition. This asymmetry is not attributable to α or α″ due to peak location; further, while the ω-phase does show peak overlap with β, splitting/asymmetry is visible in all ageing conditions, whereas reflections attributable solely to ω are not noted outside of the 12 and 24 hr conditions.

In addition to XRD, microscopic analysis was also performed using optical microscopy. In the AC condition, equiaxed grains of approximately 210 µm diameter were noted. These were predominantly β phase, though a small amount of needle-like martensitic traces were seen growing from the β-grain boundaries in limited areas, resembling prior literature on similar alloy compositions^13^.

Among the solution-treated and aged samples, few features of note could be observed, and little difference was seen between samples. Grains remained equiaxed, however mean diameter increased to ~300 µm for all aged samples, with no statistically significant variation noted regardless of ageing duration. No secondary phases were observed via this technique for any aged sample, although some dendritic structure was revealed by etching; this was not prominent in all ageing conditions, but this may simply be a function of variation in etching extent.

**Figure 3:**
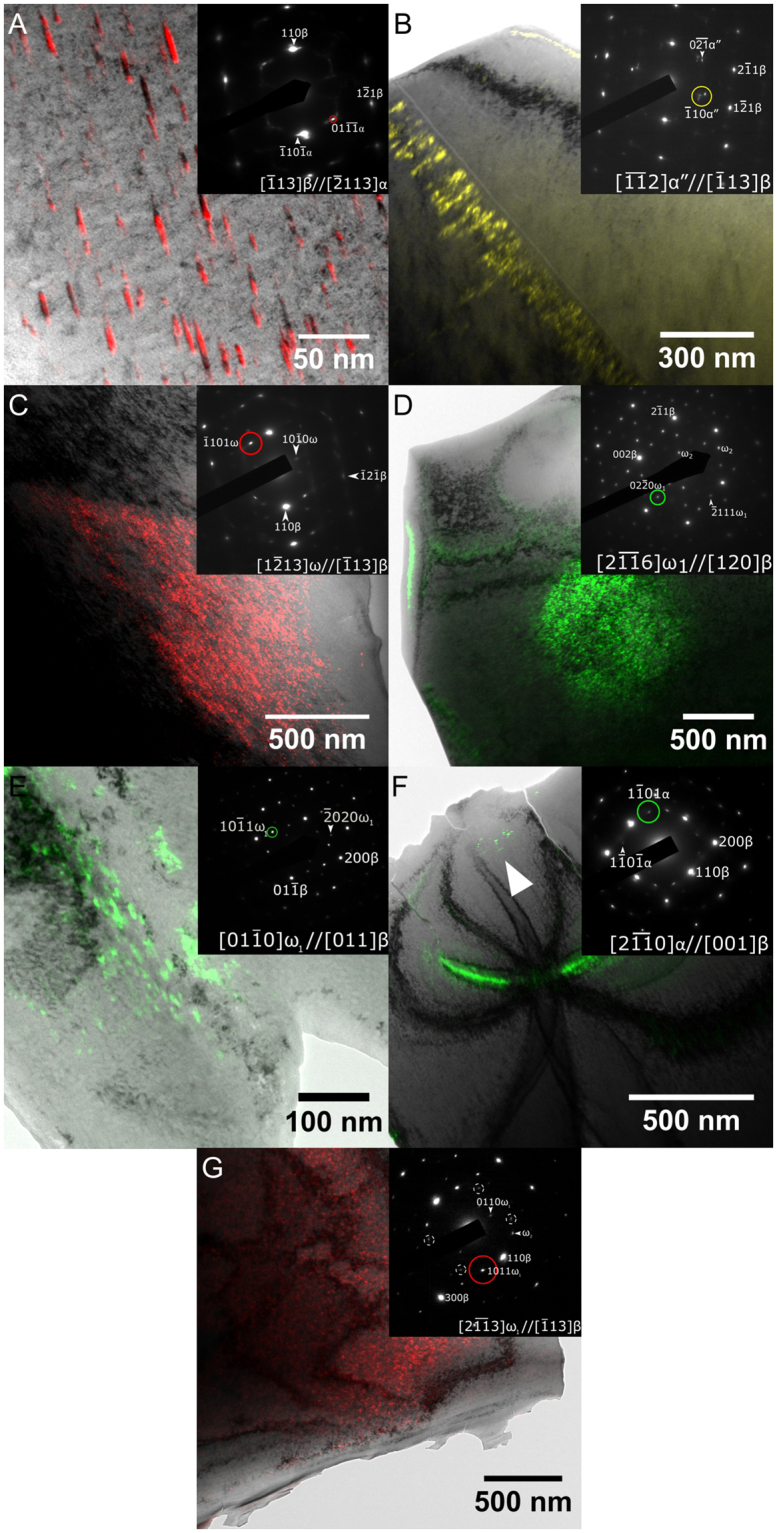
TEM micrographs. Representative micrographs for each alloy are given in (**A**–**G**). (**A**) AC (**B**) 15 min (**C**) 1.5 hr (**D**) 3 hr (**E**) 6 hr (**F**) 12 hr (**G**) 24 hr SAED patterns coinciding to imaged region displayed inset. Darkfield imaging of phases of interest are overlain in color; source reflections are circled with approximate location of Objective aperture. Please note; bright band of green contrast in F is suspected to be due to sample distortion, actual precipitates are indicated by arrow. Reflections marked with dashed circles in G are tentatively assigned to signal from a nearby folded region of sample diffracting along the [111]_β_ axis.

**Figure 4:**
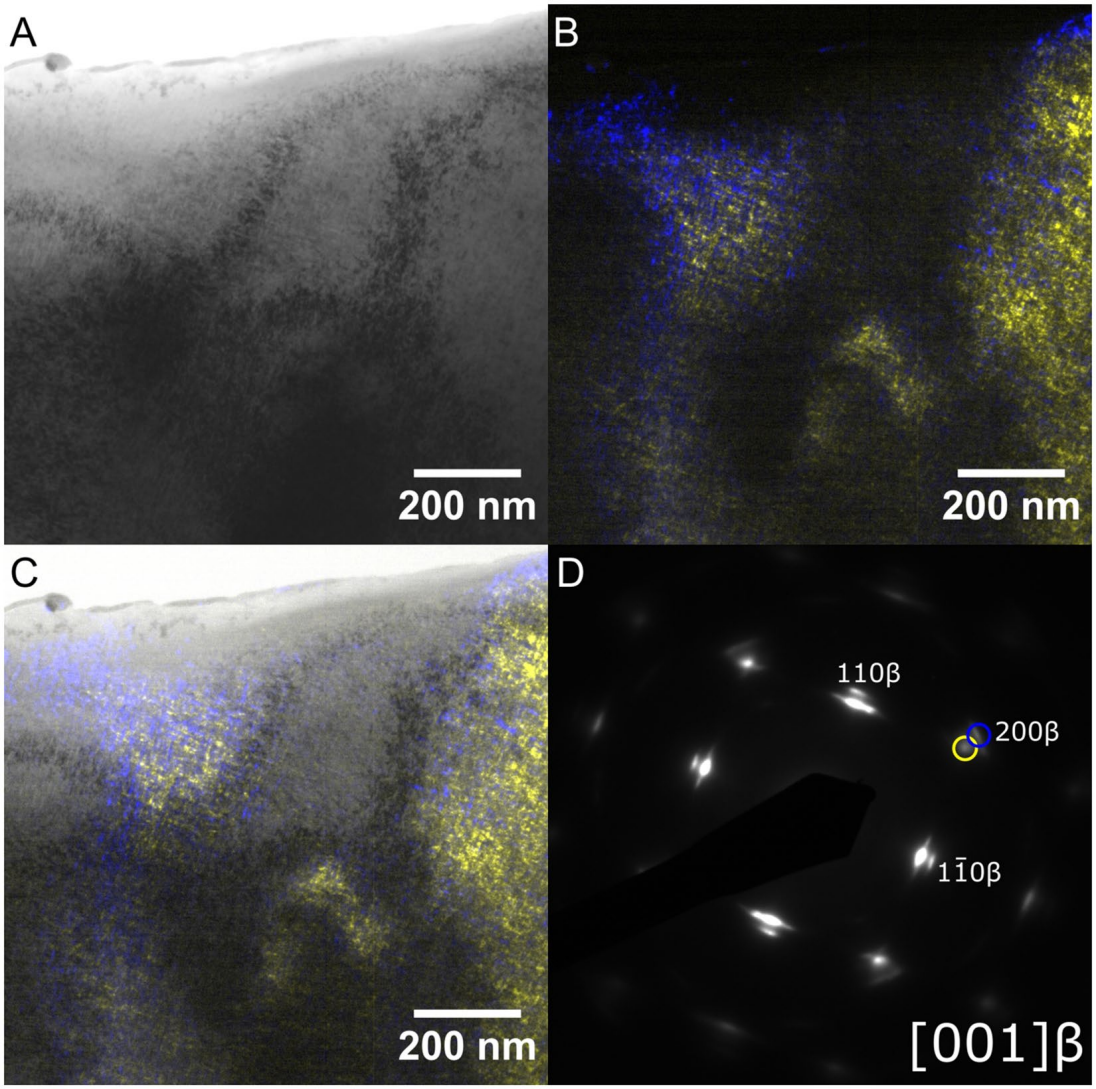
Additional TEM Micrographs. Micrographs taken from AC alloy. (**A**) Brightfield (**B**) Darkfield (**C**) Combined Brightfield and Darkfield (**D**) SAED Pattern. Darkfield imaging of phases of interest shown in colour; source reflections are circled in respective colours in D.

Further analysis was performed via transmission electron microscopy (TEM), images from which are presented in Figs 3A–G and 4A–D; darkfield imaging of reflections of interest are overlain in color, with the indicative selected area aperture position circled on the inset selected area electron diffraction (SAED).

As with XRD, the predominant phase observed under TEM in all ageing conditions was the BCC β-phase, although the other precipitate phases noted under XRD could again be observed throughout the investigated material, depending on the thermal processing.

The ω-phase was the most prominent precipitate phase, clearly present in all bar the AC and 15 min condition, and obeyed the known orientation relationship for ω formed from collapse of the β-phase, with ${[012]}_{{\rm{\beta }}}//{[01\bar{1}2]}_{{\rm{\omega }}}$ and ${(\bar{1}2\bar{1})}_{{\rm{\beta }}}\parallel {\{10\bar{1}0\}}_{{\rm{\omega }}}$^12,14^; examples are given in Fig. 3C,F,E and G. These precipitates were predominantly observed with a diameter of ~5 nm, but particle sizes of up to 20 nm were noted in the higher ageing conditions.

Beyond this, additional maxima could be observed that could be indexed to the orthorhombic α″ and HCP α. An example of the α″ phase can be observed within the twinning band in Fig. 3B; this phase was noted in the 15 min condition, and displayed orientation relationships with the parent β in good agreement with existing literature^15,16^.

Features consistent with a HCP α phase could also be resolved under SAED with either the Burgers or Pitsch-Schrader orientation relationships^17^; examples are given in Fig. 3A and F. With the exception of the AC state, precipitation was more limited than that noted for the other phases, and it was not detected at all in the 24 hr state. While acicular precipitates in the AC material were noted with lengths of 10–40 nm, these precipitates decreased in size following heat treatment, forming rounded particles with diameters on the order of 5 nm.

Finally, segregation of the β-phase could be resolved, an example of which is given in Fig. 4A–C, with attendant SAED diagram in Fig. 4D. These dense cuboidal features were faintly resolvable in the AC material under brightfield illumination (Fig. 4A), but darkfield imaging taken from the two (200)β reflections (alone in Fig. 4B, and overlain with brightfield in 5c) more clearly shows the segregation. These precipitates show an approximate width of 5–8 nm, and extend along the elastically soft <100> β directions.

After heat treatment, this cuboidal morphology could no longer be resolved; although similar splitting in the SAED could be identified in a limited number of other conditions, morphology where resolvable was spherical or ovoid particles on the order of 5 nm in diameter.

### Mechanical Analysis

**Figure 5:**
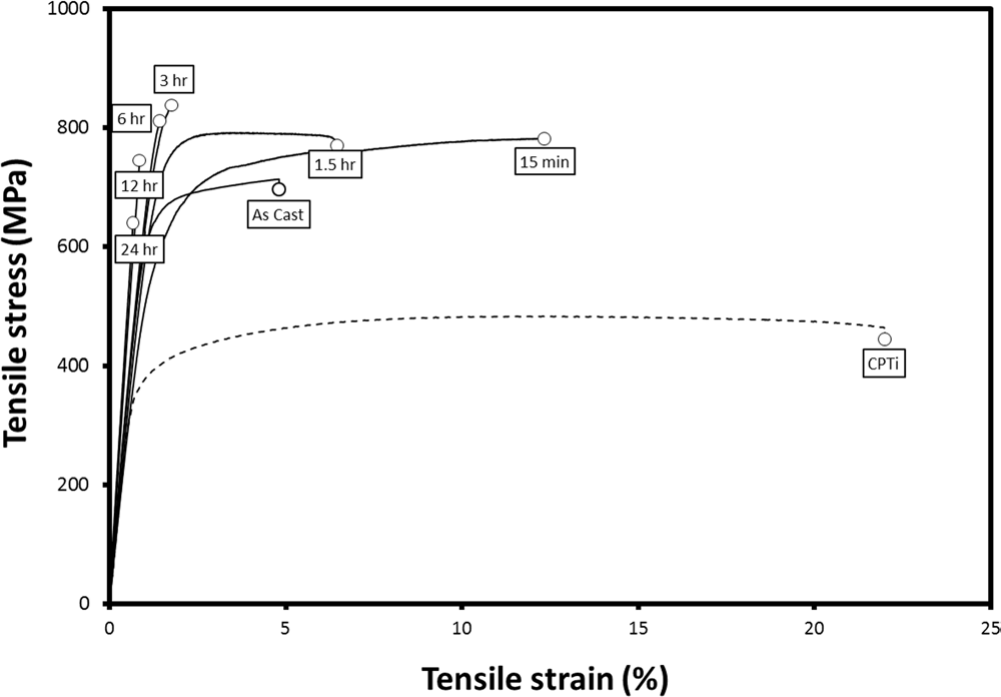
Mechanical Results. Representative tensile curves from investigated alloys.

Mechanical results were measured through two techniques in parallel. First and foremost, tensile analysis was performed on the samples. Representative tensile curves for the different ageing regimes are provided for each composition in Fig. 5, with key mechanical properties for both analytical techniques provided in Table 1.

**Table 1:** Summary mechanical properties of investigated ageing conditions.

	σ_TYS_ (MPa)	σ_UTS_ (MPa)	*E*_T_ (GPa)	δ_T_ (%)	Elongation (%)	*H*_ni_ (GPa)	*E*_ni_ (GPa)
CPTi	353 ± 5	463 ± 3	97 ± 7	0.4 ± 0.1	20.9 ± 0.2	2.9 ± 0.2	107 ± 4
AC	610 ± 30	707 ± 30	65 ± 3	0.934 ± 0.008	5 ± 2	3.8 ± 0.1	72 ± 1
15 m	550 ± 20	760 ± 10	53 ± 1	1.05 ± 0.03	13 ± 3	4.4 ± 0.2	69 ± 1
1.5 h	680 ± 10	800 ± 20	63 ± 1	1.09 ± 0.03	4 ± 2	4.4 ± 0.1	66 ± 2
3 h	770 ± 20	840 ± 30	67 ± 4	1.16 ± 0.07	1.9 ± 0.3	4.4 ± 0.3	74 ± 3
6 h	820 ± 50	830 ± 40	63 ± 3	1.30 ± 0.07	1.6 ± 0.3	5.2 ± 0.4	81 ± 5
12 h	710 ± 60	710 ± 60	86 ± 3	0.82 ± 0.05	0.82 ± 0.05	5.7 ± 0.4	96 ± 8
24 h	400 ± 200	400 ± 200	97 ± 2	0.4 ± 0.2	0.4 ± 0.2	6.2 ± 0.3	97 ± 6

Note: Values presented to first uncertain digit. Errors represent 1 Std. Dev.

Substantial changes in strength, modulus and elongation at failure occur in this alloy system with ageing. In the AC state, the material showed tensile yield (σ_TYS_) and ultimate tensile strengths (σ_UTS_) of 610 and 710 MPa respectively, as well as an elastic modulus (*E*) of 65 GPa and elongation until rupture of ~5%. Upon solution treatment and ageing, however, this behavior changed substantially; σ_TYS_ and *E* fell to 550 MPa and 53 GPa respectively in the 15 min condition, while σ_UTS and_ elongation increased sharply, up to 740 MPa and 13%, respectively.

Subsequent ageing resulted in σ_TYS_ and σ_UTS_ increasing gradually up to the 3–6 hr conditions, with maximum values on the order of 830 and 840 MPa respectively, while *E* remained approximately constant at ~65 GPa between 1.5 and 6 hrs. Ageing beyond this point, *E* spikes sharply and the measured elongation falls below 1%, with this loss of ductility adversely affecting observed strengths due to brittle fracture of the test coupon while still in the elastic region. Fracture analysis performed on these tensile specimens echo this evolution, with fracture surfaces initially dominated by fine (1–15 µm), equiaxed dimpling, that gradually transition to surfaces dominated by cleavage planes showing river markings in ageing conditions beyond 6 hrs. That said, even in the 24 hr aged state, dimpling was still apparent, and indeed fracture surfaces often transitioned between cleavage and dimpled failure without clear boundary.

Additional mechanical analysis was attempted through nanoindentation; key values are again given in Table 1. As with tensile analysis, the results show good agreement with that expected from microstructural analysis; for instance, both hardness (*H*) and modulus (*E*_ni_) show substantially higher values in the specimens displaying sufficient precipitation to appear under XRD. The measured *E* falls slightly with initial ageing before rising once more to a maximum in the 24 hr condition, in agreement with the tensile analysis, although the plateau in *E*_T_ from 1.5–6 hrs ageing time followed by a rapid increase is not reflected in *E*_NI_, with *E*_NI_ showing a more uniform increase over the same range. *H* similarly shares a trend in behaviour with σ_UTS_ initially, although differences are noted in the higher-ageing time materials, with their brittle failure behaviour.

### Biological Analysis

**Figure 6:**
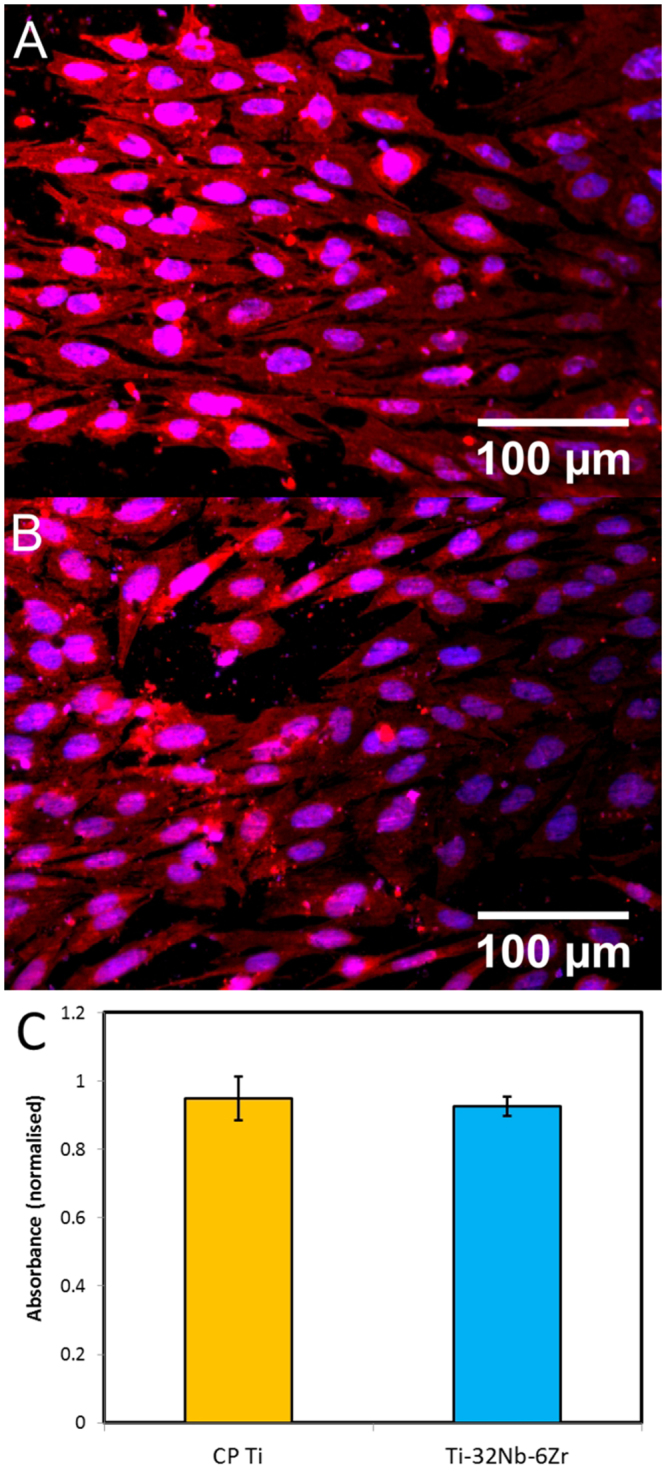
Biological analysis. (**A**) CP Ti control confocal micrograph (**B**) TNZ alloy confocal micrograph (**C**) MTS Assay. Note, vertical axis is normalised to empty-well control.

Finally, preliminary biological analysis was also conducted on the 15 min material. Representative figures from 3 and 7 days after seeding are provided in Fig. 6A,B for C.P. Ti, and c and d for the TNZ alloy, respectively. No notable differences could be seen between the control and investigated alloy, as cells seeded to both substrates primarily displayed an extended, flattened cytoplasm, with a preferred orientation parallel to the grinding direction from sample polishing. In all cases, nuclei showed a clean ovoid shape, with no signs of nuclear condensation or fragmentation.

Additional colorimetric assay data is provided in Fig. 6C, which shows no statistical difference between the C.P. Ti control and the investigated alloy.

## Discussion

While acicular α could be resolved in the AC material, likely due to the slower cooling rates of this composition, the microstructure of the alloy subsequent to solution treatment and minimal shows good agreement with that expected for the composition; the alloy displays a primarily β-phase crystal structure, with orthorhombic α″ present in detectable quantities, and no sign of the ω-phase in this condition. With further ageing, however, the investigated alloy rapidly deviated from this microstructure.

Several differences are noted; the first is the absence of the α″ phase, which suggests that further ageing triggers an α″ → β transition, matching behavior reported in similar systems^18^. Precipitation of the ω phase was also observed, occurring throughout all samples bar the AC and 15 min conditions under TEM; this is substantially distinct from XRD, where it was noted only in the 12 and 24 hr condition. Along with the similar discrepancy for α-phase, which was observed to a limited extent in all bar the 24 hr case under TEM, but noted only in the 6 hr state under XRD, this discrepancy can likely be attributed to the small crystallite size and volume fraction in the earlier ageing conditions falling below the minimum sensitivity of the utilized XRD instrument; similar behavior has been noted in prior works by the author^19^.

This small crystallite size is interesting, however; while extremely fine ω-phase precipitation in Ti is expected^12,19^, α-phase is not so restricted, and more typically forms larger acicular particles, as in the AC condition^20^. Thus, it is of note that the α-phase was seen only in a nanoscaled form following solution treatment and ageing, and constrained to limited regions. The limited presence of this phase may be due to compositional fluctuations, such as that implied by the dendritic morphology seen under OM. Given the relatively robust β-stabilisation selected, initial nucleation of this phase would be limited to those regions with suppressed Nb contents, and the growth would be limited to the initial cooling period, on the order of seconds; further growth would then be hindered by the relatively low ageing temperatures used, yielding low rates of atomic diffusion, if possible at all; notably, in work done on a similar system, Ti-32Nb-2Sn^21^, α-precipitation is not favoured at these temperatures, with ageing at 300 °C yielding no appreciable change to XRD analysis, and the α-phase instead only precipitating when aged at temperatures of 600 °C. Care should be taken in this interpretation, however, given the differing use of Sn versus Zr in the literature and present works; Sn would be expected to act as a weak β-stabiliser versus the destabilising Zr in this region of the alloy composition-space given both elements’ known effect on the $\overline{Bo}$ and $\overline{Md}$ of Ti-based alloys^10^. While the presence of α under XRD analysis in the 6-hr state suggests that some degree of α-growth during ageing lead to an increase in α volume fraction, this was difficult to confirm via TEM, and the outright absence of notable α-phase in the maximally aged state indicates some form of either α → ω or α → β with a subsequent β → ω transition occurred. This is the reverse of situations previously reported in comparable Ti-Nb systems; while α-precipitation is typically only seen at higher temperatures in similar systems^18,21–24^, in those instances where it is seen it instead grows at the expense of ω, which typically precipitates prior to α^18,24^. While mechanisms that could explain the growth of ω at the expense of other phases due to both thermal and mechanical treatment (e.g. α″ or β going to ω) are well known,^18,20,24–26^, the investigated ageing temperature is lower than that expected for the α → β transition^20^, and the α → ω transition is typically reported as occurring only under high-pressure deformation^27^. That this is occurring in the present work is possibly a function of the lower ageing temperatures utilized in the present study, given the ω-phase is increasingly stable relative to α and β as temperature falls^27,28^, but further work would be needed to confirm this.

The final feature of note is the apparent splitting of the β-phase. In light of the dendritic microstructure visible under OM, and the known enrichment of Nb/depletion of Zr in the dendritic versus interdendritic material^19,29^, the observed XRD peak splitting can likely be explained as a result of insufficient homogenisation time of the alloy. Under both SAED and XRD, the stronger of the split maxima is associated with a larger lattice parameter; this would suggest that the dominant phase is relatively Zr-enriched, relative to a Zr-poor or Nb-enriched secondary phase. However, this alone cannot explain the separation seen under TEM analysis, particularly given the pronounced cuboidal nanostructure shown in Fig. 4A–C, which is strongly reminiscent of that reported in other spinodally decomposed BCC alloys^29,30^. Rather, the split phase indicated via SAED analysis is instead considered by the authors to instead reflect spinodal decomposition products, induced by the positive enthalpy of mixing between Nb and Zr in the BCC phase; this spinodal curve can be seen to arise in the Nb-Zr binary phase diagram^31^, and is similar to that identified in prior work with a Ti-Ta-Zr alloy, arising from a similar incompatibility of Ta and Zr^29^. In the present study the material typically forms fine particulate features on the order 5–10 nm width, but this decomposition is not uniform throughout the sample, instead only occurring in limited areas of the investigated TEM foils.

Given the relatively low concentration of Zr in the bulk material, the spinodal effect is not expected to occur at all in this alloy^31^. Further, in those alloys where decomposition was expected, it would occur in broad swaths rather than limited regions, given spinodal decomposition does not require specific nucleation sites but merely suitable elemental concentrations^30^. The presence only in limited areas, combined with the outright absence of spinodal products in other regions, may however be related back to those dendritic features, as this would provide larger scale elemental segregation with limited areas potentially enriched above the point at which spinodal segregation becomes possible.

It should be noted that this splitting is not ascribed to additional phases; for instance, while the presence of α precipitation obeying the ${[2\overline{11}0]}_{\alpha }//{[001]}_{BCC}$ Pitsch-Schrader relation^15^, as in Fig. 3E, could yield diffraction maxima adjacent to the selected $\langle {110}_{\beta }\rangle$ spots, this splitting is seen even in the absence of the ${\{1\bar{1}01\}}_{\alpha }$ maxima that would be expected to fall between e.g. the ${[200]}_{\beta }$ and ${[110]}_{\beta }$ planes if this phase was present, as in Fig. 4D. Similarly, while lattice strain due to e.g. bending of the edges of the TEM foil could similarly cause a comparable SAED pattern, this cannot explain the cuboidal morphology shown under darkfield illumination.

While similar features could be observed under SAED after heat treatment, the morphology showed a sharp change from cuboidal to ovoid particles after solution treatment, suggesting the spinodal phase is not resistant to the selected solution treatment parameters. The impact of further ageing, however, was difficult to identify given the generally low incidence of this phase.

While interesting, this decomposition appears to play little role in the mechanical properties of the alloy, particularly given its limited volume fraction. Rather, the observed trends in strength, modulus and elongation of the investigated materials can be adequately explained by ω-precipitation; the gradual increase in both the yield (σ_UTS_) and ultimate tensile strengths (σ_UTS_), along with *E*_T_, up to the 6 hr mark are consistent with classical precipitation strengthening as the ω-precipitate size and volume fraction increases, with the more pronounced changes in strength and modulus beginning with the onset of precipitation of sufficient size to appear under XRD analysis^20,32^. These thresholds also match the changes in the observed fracture surfaces with dimpling, classically considered a sign of ductile failure^33^, covering all surfaces in the 15 min and 1.5 hr conditions, and still represents the dominant morphology of fracture surfaces up to 6 hrs of ageing.

Further ageing sees the alloy sharply decreases in strength due to the falling ductility over the same range; this pronounced brittleness as the material transitions into the ω-rich 12 hr and 24 hr configurations in particular is expected given ω-phase’s well-known impact on Ti-alloy brittleness, especially as particulate size exceeds 10 nm^32,33^. These ageing times also see a rise in the degree of cleavage fracture surfaces on the tensile specimens, with the 12 and 24 hr states representing a near equal split between these failure mechanisms. While cleavage itself is not representative of brittleness^33^, the decrease in dimpling over this same range is suggestive. Additionally, the fact that fracture does not seem to be intergranular is expected given the ω-phase’s relatively uniform precipitation throughout the bulk material.

Nanoindentation agrees well with tensile analysis. In particular, *E*_NI_ tracks broadly with *E*_T_, although *E*_NI_ values fall approximately 15% higher in general; given the known variation between tensile and nanoindentation results in the literature, this difference is not considered significant^34^. The trend in hardness similarly matches that of σ_UTS_ in ageing conditions up to 6 hrs; however, beyond this point, the observed *H* continues to increase whereas σ_UTS_ begins to fall sharply due to the brittleness of the material. This discrepancy is likely related to the specifics of deformation, with the suppression of Mode I failure due to compressive loading by the indenter limiting routes for crack propagation compared to tensile testing, allowing *H* to continue to increase even in the face of excessive ω precipitation.

With respect to their suitability as implant materials, it can be seen that this composition is satisfactory; even the two weakest results, in the 15 min and 24 hr condition, show σ_TYS_ in excess of 400 MPa, exceeding that of the C.P. Ti control in all cases, and in the strongest condition comparable to that seen for the upper end of Ti-6Al-4V^20^. Coupled with values of *E* as low as 53 GPa, this results in admissible strains far exceeding that of human bone, as high as 1.3%.

As a further result of these strengths, coupled with the low moduli observed, the admissible strains of all bar the two longest-aged materials are substantially better than those of bone or conventional Ti-alloys such as C.P. Ti and Ti-6Al-4V, comparable to heavily cold-rolled modern alloys such as TNTZ, and more than suitable for application as metallic biomaterials^1,20,35^.

Biological analysis similarly shows promise; although *in vitro cell* culturing serves only as a preliminary analysis, the presence of flattened, highly extended cell morphologies with extended filopodia and uniform, intact nuclei are indicative of both good cell attachment, and a benign growth environment^36,37^. Likewise, the lack of a significant difference in cell viability demonstrated via colorimetric assay between the control and TNZ alloys suggests biocompatibility on par with the already accepted C.P. Ti should be achievable.

Coupled with the mechanical properties, this therefore suggests these materials are suitable for application in orthopaedic implants.

## Conclusion

From the observed microstructural and mechanical behavior of these alloys, a number of observations can be made.

- Prolonged, low-temperature ageing treatments may see dissolution of α at the expense of ω-phase growth. Given the known detrimental effects of this phase on mechanical properties, this should therefore be avoided.
- Mechanical properties suitable for application in biomedical orthopaedic implants were achieved; namely, tensile yields up to 820 MPa, and moduli as low as 53 GPa were observed, resulting in admissible strains of up to 1.3% in the 6 hr condition.
- Diffraction analysis showed apparent splitting of BCC phase. This is hypothesised to be due to spinodal decomposition of the matrix β-phase, driven by segregation of Nb and Zr, occurring in regions of Zr enrichment resulting from casting dendrites.
- Preliminary analysis of biological response further suggests that this material is comparable to the commonly used biomedical alloy, ASTM grade 2 commercially pure titanium.

## Materials and Methods

### Manufacturing

The investigated alloy was composed of Ti with 32 wt.% Nb and 6 wt.% Zr. The nominal location on a $\overline{Bo}-\overline{Md}$ phase diagram is presented in Fig. 1. The alloy was produced from 99.95% pure powders by cold-crucible levitation melting, with the resulting ingot sectioned via wire electrical discharge machining (WEDM). A portion was retained in the as-cast condition, then the remaining material was sealed in quartz tubes under vacuum and solution treated at 890 °C for 1 hr, before the tubes were broken and samples were water quenched. The material was again sealed under vacuum, and aged at 300 °C for a range of times before again being quenched; ageing times used were 15 min, 1.5 hr, 3 hr, 6 hr 12 hr and 24 hr.

Following thermal processing, samples were sectioned by WEDM, SiC and diamond saws as necessary into rods for disks for microstructural observation, and dog-bone coupons for tensile testing.

### Microstructural Analysis

Microstructural observation was undertaken via optical microscopy (OM), X-ray diffraction (XRD) and transmission electron microscopy (TEM).

For XRD, disks of 8 mm diameter and 2 mm thickness were ground to a 2400 grit finish via SiC paper, and then samples were mounted and analysed via a Bruker Axs D4 Endeavor using Cu Kα radiation. 2θ was varied from 10–90° with a step size of 0.02°.

For OM, samples of similar dimension were used, however further polishing was done using a mixture of a colloidal silica suspension (OP-S) and hydrogen peroxide until a mirror finish was achieved. Following this, the samples were etched using Kroll’s reagent until surface features could be resolved. Grain size was analysed in accordance with the Abrams Three-Circle Procedure, outlined in ASTM E-112^38^.

TEM analysis was undertaken on 3 mm foils, using JEOL-1010 and JEOL-2010 microscopes; these were prepared from disks cut via WEDM to 1 mm of thickness, then ground via SiC papers to a thickness of 80–100 µm, dimpled to a central thickness of 20 µm, and finally thinned to electron transparency (~100 nm) using a JEOL EM-09100 Ion Slicer.

### Mechanical Analysis

Mechanical analysis was performed via tensile deformation and nanoindentation techniques.

Tensile tests were performed on dog-bone specimens with a length of 100 mm, width of 10 mm and thickness of 1 mm, with gauge section length and width of 32 mm and 6 mm respectively. Tensile analysis was performed via a uniaxial 100 kN Materials Testing Systems servo hydraulic testing machine at a constant displacement rate of 1.2 mm/min, with strain measured via an extensometer. Yield stress was derived via the 0.2% offset method. Following completion of tensile tests, test coupons were imaged via a FEI Quanta 200 ESEM scanning electron microscope (SEM) under secondary electron conditions to determine fracture behavior.

Nanoindentation was performed using a Hysitron TI-950 TriboIndenter with a Berkovich diamond tip. A total of 27 indents were taken as three sets of nine indents from different regions per ageing condition, using a 5 mN load force, 10 s loading, dwell, and unloading times. Elastic moduli derived from nanoindentation (E_ni_) were calculated in accordance with the Oliver-Pharr method^39^, with the Poisson ratio (v) for the Ti-Nb-Zr alloy approximated as 0.344; this was derived from literature values for Ti-Nb binary alloys of equivalent $\overline{e/a}$ ratio and Nb-content^8^, as Zr is known to have minimal effect on E, G and thus v when substituted for Ti^40^.

For comparison with all mechanical tests, commercially pure ASTM Grade 2 Ti (CP-Ti) in the cold-rolled and annealed state was similarly analysed.

### Biological Analysis

In addition to mechanical and microstructural characterisation, a preliminary analysis of the biological response to the investigated alloy was undertaken, using human osteoblast-like sarcoma cells cultured *in vitro*. This method was performed in accordance with that outlined in previous work^29,36^; SaOS-2 cells were seeded on disc specimens polished to a 600 grit finish in a 48-well plate, using empty wells for a negative control and commercially pure Ti (grade 2 ASTM) plates as a positive control, at a density of 1 × 10^4^ cells per well and 200 µL of modified minimum essential medium (MMEM, composition given in^36^), then incubated at 37 °C for 1 h under an atmosphere of humidified 5% CO2 in air (all further incubation steps performed under these conditions), after which a further 500 µL of MMEM was added. Incubation was then continued for 7 days, after which cells were fixed, stained with phalloidin red and 40,6-diamidino-2-phenylindole (DAPI) fluorescent dyes, and then imaged using confocal microscopy (CM) (Olympus IX81). Concurrently, SaOS-2 cell proliferation was also measured through colorimetric assay utilizing the reduction of a tetrazolium salt (MTS), again in accordance with the previous work^29,36^; specimens were seeded as with the confocal specimens, incubated in the initial 200 µL of MMEM for 24 h, then the MMEM was replaced by a new 200 µL aliquot and incubation continued for a further day. The control medium in each well was then replaced by 150 µL of phenol-red-free minimum essential medium, and 50 µL of a MTS/phenazine methosulfate solution was added to each well, then further incubated for an additional hour. 100 µL aliquots were then taken, and absorbance was measured at 490 nm via photospectrometer.

For both confocal and colorimetric analysis, at least 3 separate samples were analysed for each of the TNZ and CP Ti alloys.

### Statistical analysis

All error bars presented throughout this document represent standard deviation unless otherwise noted.

Where appropriate, statistical analysis was performed via use of single factor ANOVA, with p = 0.05. Where significance was noted, pairwise 1-sided Student T-tests were performed as a follow-up.

### Data availability

The datasets generated during and/or analysed during the current study are available from the corresponding author on reasonable request.
